# Clinicopathological Features of Patients Nominated for Head and Neck Biopsies: A One-Year Series

**DOI:** 10.7759/cureus.13666

**Published:** 2021-03-03

**Authors:** Fawaz D Alshammari, Samir Abdulkarim Alharbi, Mohamed Ahmed B Bealy, Khalid Abd Elmohsin Awad Elseed Idris, Ali Ahmed Alqahtani, Hussain G Ahmed

**Affiliations:** 1 Department of Clinical Laboratory, College of Applied Medical Science, University of Hail, Hail, SAU; 2 Department of Clinical Laboratory, College of Applied Medical Science, Shaqra University, Shaqra, SAU; 3 Department of Pathology, College of Medicine, University of Hail, Hail, SAU; 4 Department of Pathology, King Salman Hospital, Hail, SAU; 5 Department of Pathology, King Khalid Hospital, Hail, SAU; 6 Department of Histopathology and Cytology, Faculty of Medical Laboratory Sciences, University of Khartoum, Khartoum, SDN; 7 Department of Pathology, University of Hail, Hail, SAU

**Keywords:** head and neck, benign, saudi arabia, lesions

## Abstract

Background

Head and neck lesions, which are predominantly benign, were widely reported. Some of these tumors are potentially neoplastic and others are non-neoplastic. Therefore, this study aimed to assess the clinicopathological features of patients nominated for head and neck biopsies.

Methodology

In this study, data regarding head and neck biopsies were retrieved from the Department of Pathology at King Khalid Hospital, Hai'l, Northern Saudi Arabia. Data referring to head and neck biopsies of patients who were diagnosed during the period from January 2018 to December 2018 were included.

Results

The initial clinical presentations were stated for 50/64 (78.1%) head and neck lesions, 12/64 (18.8%) head and neck cysts, 1/64 (1.6%) keloid, and 1/64 (1.6%) ischemia. With regard to the biopsy's site, most were taken from the nose followed by oral cavity, scalp, ear, face, and eye, constituting 19/64 (29.7%), 15/64 (23.4%), 9/64 (14.1%), 5/65 (7.8%), 4/64 (6.2%), and 3/64 (4.7%), respectively.

Conclusion

Head and neck benign lesions, predominantly inflammatory lesions, are common in Northern Saudi Arabia. Accurate identification of these lesions is important during histopathological diagnosis, as some have pathological features that mimic some potentially neoplastic lesions.

## Introduction

Head and neck cancers (HNCs) represent a group of heterogeneous malignancies that are responsible for diverse cancer-related morbidities and mortalities worldwide. These cancers are related to numerous exposures to specific etiological factors, which usually determine the pattern of subsequent prognosis [1]. In recent years, there is an increasing incidence of HNCs, which is attributed to the prevalent existence of high-risk human papillomavirus (HPV) subtypes, besides the consumption of tobacco and alcohol in numerous communities [2,3].

Many benign head and neck lesions were widely reported. Some of these tumors are potentially neoplastic and others are non-neoplastic [4,5]. Consequently, the overall management of any head and neck tumor is deemed important for better patient outcomes.

However, there is a paucity of data regarding the epidemiology of HNCs from Saudi Arabia. Some studies from certain regions in Saudi Arabia have shown high prevalence rates of HNCs [6]. Although HNCs have bad outcomes, early detection through the implementation of prevention and control programs plays a major role in offering better outcomes and best prognosis [7]. These can be achieved by implementing educational programs as well as raising population awareness [8]. Usually, poor awareness may result in late diagnosis, poor prognosis, and eventually poor treatment outcomes [6]. As a result, we investigated a group of patients with head and neck lesions to evaluate the burden of potential neoplastic lesions in Northern Saudi Arabia. Therefore, this study aimed to assess the clinicopathological features of patients nominated for head and neck biopsies.

## Materials and methods

In this study, data regarding head and neck biopsies were retrieved from the Department of Pathology at King Khalid Hospital, Hai'l, Northern Saudi Arabia, during the period from January 2018 to December 2018. The diagnosis of head and neck lesions was confirmed by conventional histopathology. The re-evaluation of the histopathological diagnosis of the tissue samples was completed to confirm the prior diagnosis and to categorize the classification of the lesion into benign or malignant types.

Statistical analysis

Obtained information sets were entered into Statistical Package for Social Sciences (SPSS) Version 16 (SPSS Inc., Chicago, IL, USA). The chi-square test was employed to assess the statistical significance (p < 0.05 was considered significant).

 Ethical consent

The protocol of this study was established agreeing with the 2013 Declaration of Helsinki, and this study was approved by the Ethics Committee of the College of Medicine, University of Hail, Hai’l, Saudi Arabia.

## Results

This series of patients included 64 patients who underwent head and neck biopsies for histopathology diagnosis. The patients included 31(48.4%) males and 33(51.6%) females, aged 3 to 79 years, with a mean age of 34 years.

**Figure 1:**
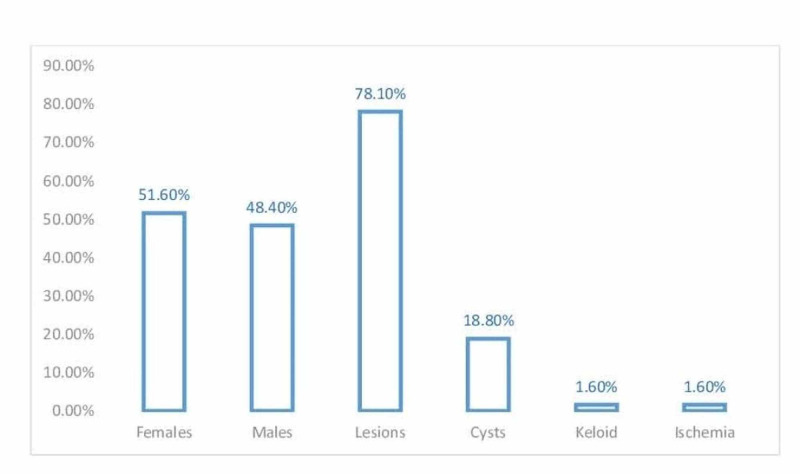
Description of patients by sex and initial clinical presentation

The initial clinical impression was stated for 50/64 (78.1%) head and neck lesions, 12/64 (18.8%) head and neck cysts, 1/64 (1.6%) keloid, and 1/64 (1.6%) ischemia, as shown in Figure 1.

**Figure 2:**
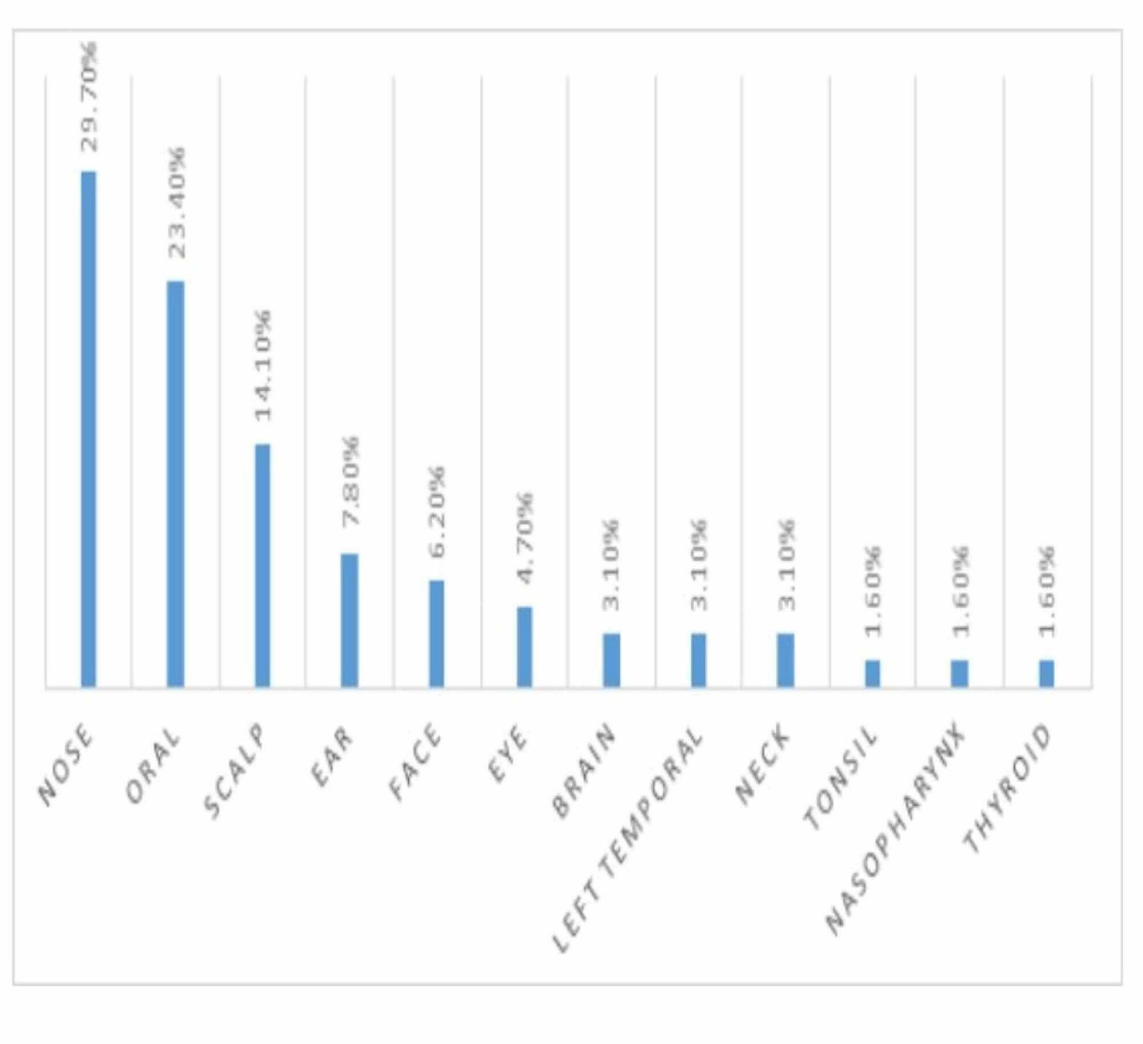
Description of patients by biopsy site

Concerning the biopsy's site, most were taken from the nose followed by oral cavity, scalp, ear, face, and eye, constituting 19/64 (29.7%), 15/64 (23.4%), 9/64 (14.1%), 5/65 (7.8%), 4/64 (6.2%), and 3/64 (4.7%), respectively as indicated in Figure 2.

**Figure 3:**
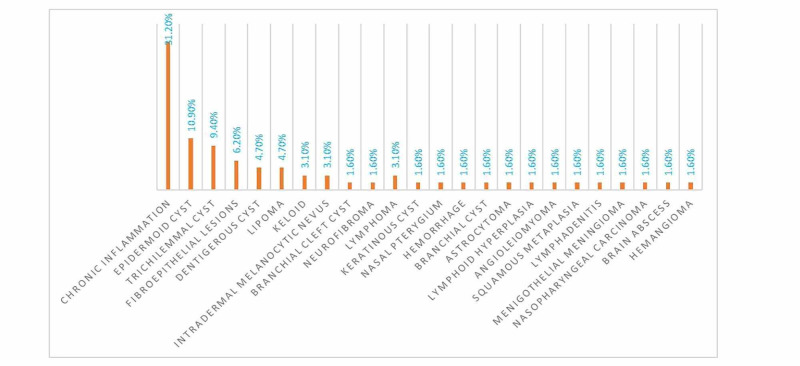
Description of patients by diagnosis

A number of these biopsies were characterized as chronic inflammation (20/64 [31.2%]) followed by epidermoid cysts (7 [10.9%]), trichilemmal cysts (6 [9.4%]), fibroepithelial lesions (4 [6.2%]), and others, as indicated in Figure 3.

**Figure 4:**
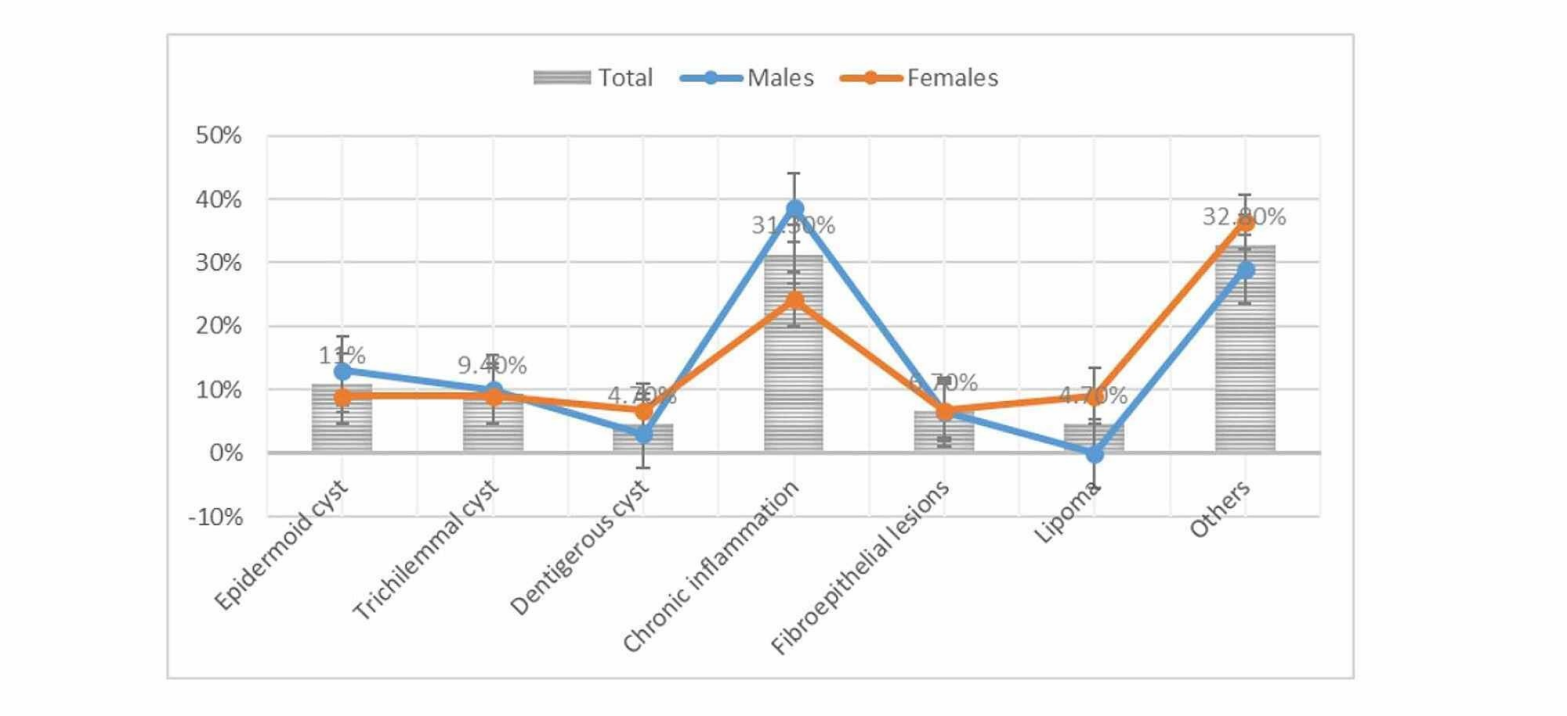
Percentages of diagnosis by sex

The data showing the relationship between diagnosis and sex are shown in Table 1 and Figure 4. Among those diagnosed with chronic inflammation, 12/31 (38.7%) were males and 8/33 (24.2%) were females. Epidermoid cysts were diagnosed in 4/31 (13%) males and 3/33 (9%) females. All cases of lipoma were seen among females.

**Table 1:** Diagnosis by sex

Diagnosis	Males	Females	Total
Epidermoid cyst	4	3	7
Trichilemmal cyst	3	3	6
Dentigerous cyst	1	2	3
Chronic inflammation	12	8	20
Fibroepithelial lesions	2	2	4
Lipoma	0	3	3
Others	9	12	21
Total	31	33	64

**Figure 5:**
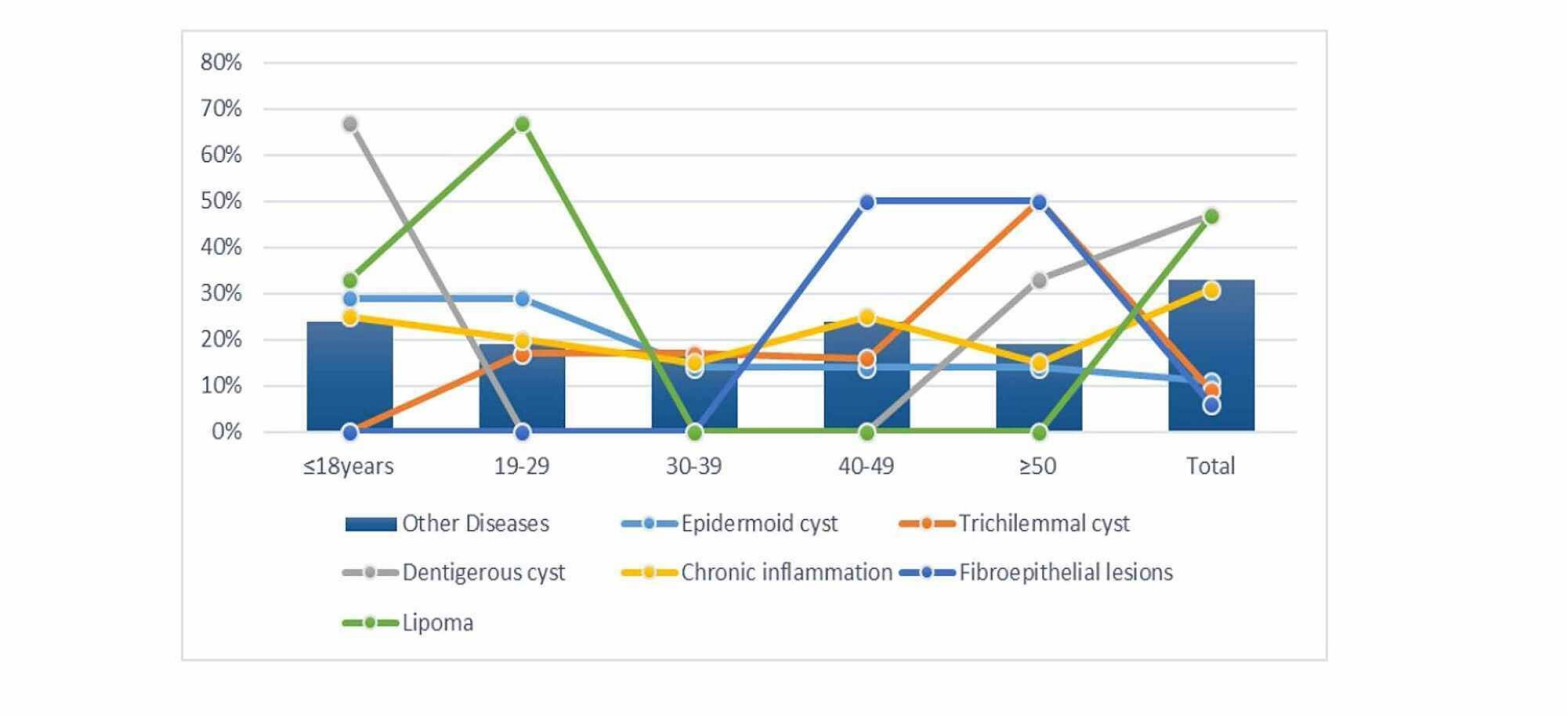
Diagnosis by age within the entire pathological condition

As summarized in Table 2 and Figure 5, epidermoid cyst, trichilemmal cyst, dentigerous cyst, chronic inflammation, and lipoma was frequently seen in under the age of 29 years, ≥50 years, ≤18 years, <29 years, ≥40 years, and <29 years, in this order, representing 4/7 (57%), 3/6 (50%), 2/3 (67%), 9/20 (45%), 4/4 (100%), and 3/3 (100%) patients, respectively.

**Table 2:** Diagnosis by age

Diagnosis	≤18 years	19-29 years	30-39 years	40-49 years	≥50 years	Total
Epidermoid cyst	2	2	1	1	1	7
Trichilemmal cyst	0	1	1	1	3	6
Dentigerous cyst	2	0	0	0	1	3
Chronic inflammation	5	4	3	5	3	20
Fibroepithelial lesions	0	0	0	2	2	4
Lipoma	1	2	0	0	0	3
Others	5	4	3	5	4	21
Total	15	13	8	14	14	64

**Figure 6:**
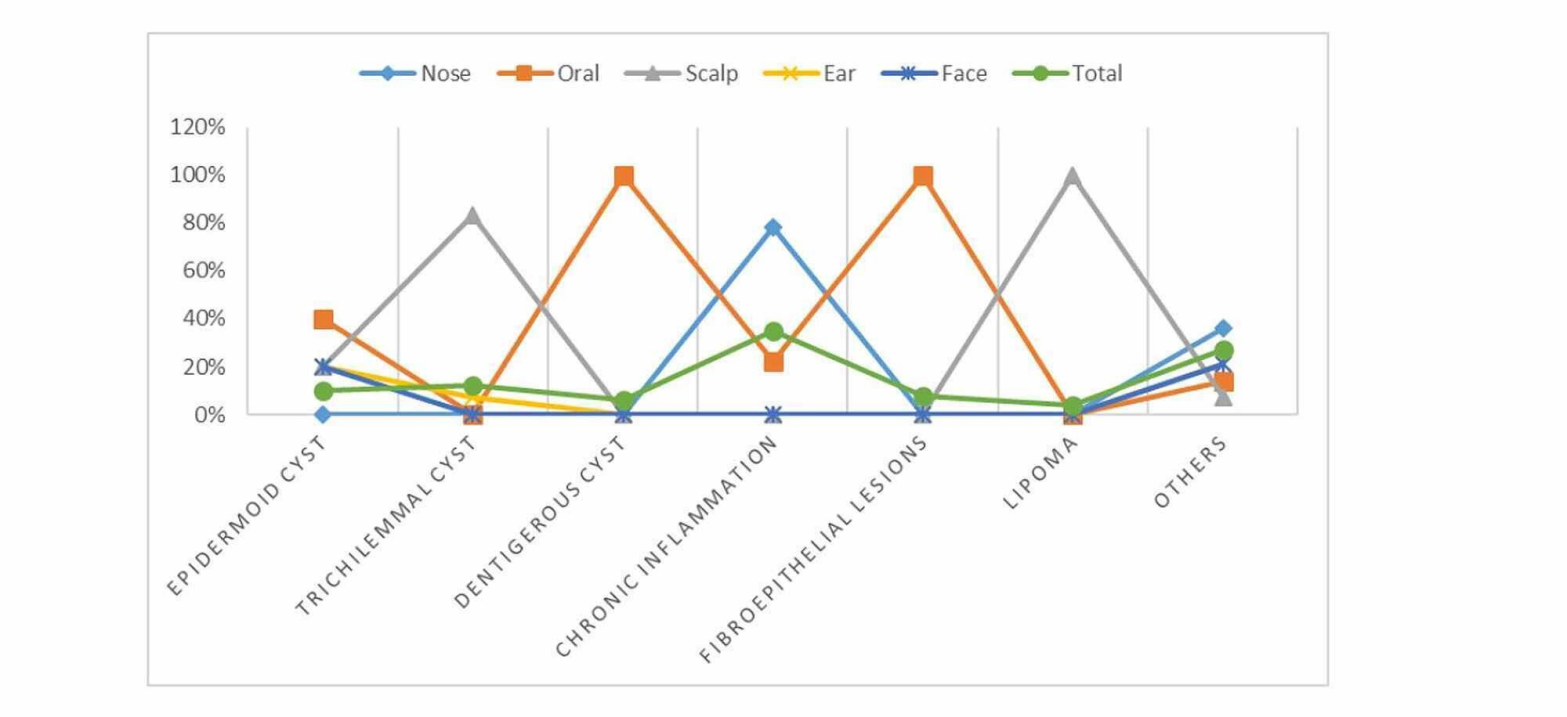
Diagnosis by the most common head and neck sites within the entire disease group

The common anatomical sites for epidermoid cyst, trichilemmal cyst, dentigerous cyst, chronic inflammation, and lipoma were oral, scalp, oral, nose, oral, and scalp, in that order, constituting 2/5 (40%), 5/6 (83%), 3/3 (100%), 14/18 (78%), 4/4 (100%), and 2/2 (100%), respectively, as shown in Table 3 and Figure 6.

**Table 3:** Diagnosis by the most common head and neck sites

Diagnosis	Nose	Oral	Scalp	Ear	Face	Total
Epidermoid cyst	0	2	1	1	1	5
Trichilemmal cyst	0	0	5	1	0	6
Dentigerous cyst	0	3	0	0	0	3
Chronic inflammation	14	4	0	0	0	18
Fibroepithelial lesions	0	4	0	0	0	4
Lipoma	0	0	2	0	0	2
Others	5	2	1	3	3	14
Total	19	15	9	5	4	52

As shown in Table 4, most conditions presented in the form of lesions (50/62 [81%]) and few in the form of cysts (12/62 [19%]). Frequent cysts clinical presentations were in epidermoid cyst, trichilemmal cyst, and dentigerous cyst conditions, representing 4/7 (57%), 3/6 (50%), and 2/3 (67%), respectively.

**Table 4:** Diagnosis by the most common clinical presentations

Diagnosis	Cysts	lesions	Total
Epidermoid cyst	4	3	7
Trichilemmal cyst	3	3	6
Dentigerous cyst	2	1	3
Chronic inflammation	1	19	20
Fibroepithelial lesions	0	4	4
Lipoma	0	3	3
Others	2	17	19
Total	12	50	62

## Discussion

HNCs represent one of the major health problems. Consequently, in this study, we investigated a series of patients with head and neck lesions to explore their potentiality, as some have pathological features that mimic some potentially neoplastic lesions.

In this study, around 78.1% of the patients presented with clinically non-specific head and neck lesions. As head and neck lesions, most of these lesions happened in the nose followed by oral cavity and scalp, constituting 29.7%, 23.4%, and 14.1%, respectively. Oral and maxillofacial sites are the most frequent sites for head and neck lesions, as widely reported [9].

Around 31.2% of patients in this study were diagnosed with chronic inflammation. Majority of the cases were found in the nose and oral cavity. Several conditions have been identified that induce inflammatory events in head and neck sites, particularly the oral cavity [10]. Moreover, most head and neck potential neoplastic and malignant tumors tend to promote inflammatory reactions [11]. Thus, these findings might be attributed to the lesion potentiality. Moreover, the common exposure of some head and neck sites such as oral and nasal cavities to the inflammatory conditions, besides the possibility of HPV infection can also support these findings [11-13]. Moreover, inflammatory changes were more common among males and those under 30 years. However, there is a lack of data regarding the correlation between head and neck inflammatory changes and age or sex. Most of these inflammatory lesions were seen in the nose (77.8%) followed by oral site (22.2%). The nose is frequently affected by non-neoplastic inflammatory lesions, which are categorized into infectious conditions, chronic rhinosinusitis, and autoimmune conditions [14]. Other inflammatory conditions of the nose include granulomatous vasculitis Wegener's, sarcoidosis, relapsing polychondritis, and nasal perforation [15].

All of the fibroepithelial lesions were found in the oral cavity and among patients over 40 years. Several studies have reported that fibroepithelial hyperplasia is the most frequent oral non-neoplastic lesion, particularly fibroepithelial polyp [16,17]. The link of these lesions in the older population was previously supported in the literature [18].

Around 83% of the trichilemmal cysts were found in the scalp and the remaining 17% in the ear site. Although this cyst can occur in head and neck sites, it is commonly reported to be on the scalp [19].

With regard to the clinical presentation of the patients in this study, 57% of the patients with epidermoid cyst presented with lesions in form of cysts, while the remaining 43% presented in form of lesions. The epidermoid cyst can present in different forms mimicking sold lesions [20] and can occur in different parts of the body [21].

Nevertheless, most (81%) of the benign head and neck lesions present in the form of solid lesions and only 19% present in the form of cysts. However, the everyday practice supports the findings of this study that most head and neck tumors present in the form of solid lesions rather than cysts, particularly inflammatory lesions, which represent the majority in this study.

Although our study provided useful information regarding head and neck benign lesions, it has some limitations including its retrospective setting and lack of some biological indicators due to the absence of direct patient assessment.

## Conclusions

Head and neck benign lesions, predominantly inflammatory lesions, are common in Northern Saudi Arabia. Accurate identification of these lesions is important during histopathological diagnosis, as some have pathological features that mimic some potentially neoplastic lesions.
